# Application of a Handheld Near-Infrared Spectrometer to Predict Gelatinized Starch, Fiber Fractions, and Mineral Content of Ground and Intact Extruded Dry Dog Food

**DOI:** 10.3390/ani10091660

**Published:** 2020-09-16

**Authors:** Arianna Goi, Marica Simoni, Federico Righi, Giulio Visentin, Massimo De Marchi

**Affiliations:** 1 Department of Agronomy, Food, Natural Resources, Animals and Environment, University of Padova, Viale dell’Università 16, 35020 Legnaro (PD), Italy; arianna.goi@unipd.it; 2 Department of Veterinary Science, University of Parma, Via del Taglio 10, 43126 Parma, Italy; marica.simoni@unipr.it (M.S.); federico.righi@unipr.it (F.R.); 3 Department of Veterinary Medical Sciences, Alma Mater Studiorum-University of Bologna, Via Tolara di Sopra 50, 40064 Ozzano dell’Emilia (BO), Italy; giulio.visentin@unibo.it

**Keywords:** dog food, handheld spectrometer, minerals, NIR spectroscopy, starch

## Abstract

**Simple Summary:**

The pet food industry is interested in performing fast analyses to control the nutritional quality of their products. Despite having some limitations related to the need to modify the production process or to have a laboratory to prepare the samples for analysis through desktop instruments, near-infrared spectroscopy is one of the most used technologies for inexpensive analysis of foodstuffs. Thus, the miniaturization of infrared devices allows a wider industrial applicability of this technique. Information on the use of miniaturized infrared tools in the pet food sector is currently very limited, and the present research is the first attempt to predict the total and gelatinized starch, insoluble fibrous fractions, and mineral content of ground and intact dry pet food using the handheld NIR scanner SCiO™. Results from the current study revealed no significant differences in the predictive ability of the instrument using both ground and intact samples. The instrument offers a potential for screening purposes of both total and gelatinized starch, revealing the potential to monitor their content and ratio in commercial dog food on a large scale. Improvements such as widening the wavelength range is expected to increase prediction models’ accuracy.

**Abstract:**

The aim of the present study was to investigate the ability of a handheld near-infrared spectrometer to predict total and gelatinized starch, insoluble fibrous fractions, and mineral content in extruded dry dog food. Intact and ground samples were compared to determine if the homogenization could improve the prediction performance of the instrument. Reference analyses were performed on 81 samples for starch and 99 for neutral detergent fiber (NDF), acid detergent fiber (ADF), acid detergent lignin (ADL), and minerals, and reflectance infrared spectra (740 to 1070 nm) were recorded with a SCiO™ near-infrared (NIR) spectrometer. Prediction models were developed using modified partial least squares regression and both internal (leave-one-out cross-validation) and external validation. The best prediction models in cross-validation using ground samples were obtained for gelatinized starch (residual predictive deviation, RPD = 2.54) and total starch (RPD = 2.33), and S (RPD = 1.92), while the best using intact samples were obtained for gelatinized starch (RPD = 2.45), total starch (RPD = 2.08), and K (RPD = 1.98). Through external validation, the best statistics were obtained for gelatinized starch, with an RPD of 2.55 and 2.03 in ground and intact samples, respectively. Overall, there was no difference in prediction models accuracy using ground or intact samples. In conclusion, the miniaturized NIR instrument offers the potential for screening purposes only for total and gelatinized starch, S, and K, whereas the results do not support its applicability for the other traits.

## 1. Introduction

Product quality is one of the most important elements underpinning consumer confidence. The dog food industry pays great attention to the quality of both raw materials used in the production process and final food products in order to offer high-quality foods. Dog food products available on the market should be able to supply the digestible nutrient amounts needed to guarantee the complete provision of the animals’ nutritional requirements in each phase of their life as well as maintain their health status.

Starch constitutes up to 50% of dogs’ dry diet and represents an important source of digestible energy due to the release of glucose after its digestion. Moreover, from a technological point of view, its presence is necessary to obtain the specific honeycomb structure of kibbles [1]. Since dog foods contain a relatively large quantity of starch, it has been observed that the digestibility of the whole diet depends extensively on the digestibility of this constituent [2]. The most commonly used process for dry dog food production is extrusion, which is a thermomechanical treatment that leads to starch gelatinization [3,4]. Extrusion is responsible for a substantial modification of starch structure, with implications on its digestibility and consequently on the metabolic response of dogs to this nutrient [5]. For these reasons, the control of the ratio between gelatinized and total starch is important in order to monitor the cooking process. From a practical point of view, this ratio measures the proportion of rapidly digestible starch fraction over its total amount.

According to Van Soest et al. [6], fibrous fractions that are considered non-digestible are the neutral detergent fiber (NDF; hemicellulose, cellulose, lignin, insoluble proteins, cutin, Maillard products, tannin condensates, and insoluble minerals), the acid detergent fiber (ADF; insoluble cellulose, lignin, cutin, Maillard products, condensed tannins, and insoluble minerals), and the acid detergent lignin (ADL; lignin, cutin, Maillard products, and silica). The evaluation of the fiber fractions allows the quantification of insoluble and non-viscous components of the plant fiber which are hemicellulose, cellulose, and lignin [7]. Cellulose and lignin can be considered insoluble and unfermentable fiber, while hemicellulose is poorly fermented by non-ruminants [8]. From a physiological perspective, they represent an important part of the diet because of their association with ingesta transit time, gastric emptying rate, and feces characteristics [9,10]. Thus, their evaluation is fundamental when formulating diets.

Furthermore, for the manufacturing companies, it is important to monitor the mineral content of foods, for its impact on the animals’ health and growth [11]. Both macrominerals and trace minerals exert important functions in the maintenance of homeostasis [12,13,14], and their excess or deficiency can impair animals’ health [15]. Thus, the mineral content of dog food needs to be frequently monitored with an easy and fast method.

Near-infrared spectroscopy (NIRS) is currently one of the most widespread technologies for the rapid and inexpensive analysis of foodstuffs, and it is also widely applied in pet food factories to predict the products’ gross composition in terms of moisture, protein, and fat. Its main advantages are as follows: (i) the possibility to perform the analyses of multiple compounds simultaneously in a rapid and easy way, without compromising the sample since it is a non-destructive method; (ii) the absence of chemical products usage and thus of chemical waste; and (iii) the lower analytical costs compared to other techniques [16]. Moreover, NIRS can be used in the industry to monitor the manufacture process “in real time”. In particular, this technology can be applied in-line, at-line, or off-line by positioning the instrumentation at different steps of the production chain based on the quality control protocols, in order to take corrective actions, if needed, in advance and potentially before the production process is terminated. The in-line method allows performing a continuous process control without the need for sampling, while with at-line analysis, the samples are removed from the process and analyzed using a near-infrared (NIR) spectrometer located in a laboratory near the manufacturing chain. Finally, off-line analysis is performed in a central laboratory by qualified operators, but results are not available within a short time. At-line and off-line methods could have some limitations due to the need to apply changes to the production process or to have a laboratory in which to prepare the samples to be analyzed using a desktop instrument [17,18]. On the other hand, miniaturized NIRS is very promising for the pet food industry aiming at the control of their production processes in-line in a cost-effective manner, allowing the determination of quality control parameters rapidly and without the need for the modification of the manufacturing plant. To our knowledge, there is very limited information concerning the use of miniaturized “pocket” infrared tools in the pet food sector, and the present paper is the first attempt to predict the composition of dry pet food using the miniaturized NIR scanner SCiO™ (Consumer Physics Inc., Tel Aviv, Israel). Moreover, other researchers have reported the potential of this instrument for the prediction of energy and carbohydrate contents in drinks [19], total soluble solids, maturity in fruits [20], meat composition [21], intact casein, and total protein in cheese [22].

The objective of the present research was to investigate the feasibility of the handheld spectrometer to predict total and gelatinized starch, fibrous fractions, and mineral content in extruded dry dog food. The comparison of the predictions made on intact and ground samples aimed at determining whether homogenization could improve the predictive ability of the instrument.

## 2. Materials and Methods

### 2.1. Sample Collection

A total of 99 extruded dog food sealed commercial packages of 2, 2.5, and 3 kg were collected in a pet food factory in Northern Italy (Dorado S.r.l.; Monsole di Cona, Venice, Italy) and stored in the dark and at room temperature in the laboratory of the Department of Agronomy, Food, Natural resources, Animals and Environment of the University of Padova (Legnaro, Italy) for analysis. The dry dog food varieties used in this study were representative of the Italian pet food market in terms of composition, and they were intended for both puppy and adult dogs of small, medium, and large breed sizes. This sampling methodology was intended to maximize the ample nutrient variability necessary to develop accurate prediction models [23]. From each package, 100 g of product were ground with a knife mill to pass a 1 mm screen (Retsch Grindomix GM200; Retsch GmbH & Co, Haan, Germany) and divided in two aliquots: one for the reference analyses and the other for the handheld NIRS analysis. Moreover, the remaining kibbles were preserved to perform NIRS analysis on the intact product. Table 1 shows the chemical composition—dry matter (DM), crude protein (CP), ether extract (EE), crude ash, crude fibers, and nitrogen-free extract—provided by the manufacturer and converted on a DM basis.

### 2.2. Reference Analyses

All 99 ground samples were analyzed for macrominerals (Ca, P, Mg, Na, K, and S), trace minerals (Al, B, Ba, Cr, Cu, Fe, Li, Mn, Mo, Ni, Sr, V, and Zn), NDF, ADF, and ADL. Total and gelatinized starch were determined on 81 ground samples due to the limited number of assays that could be performed from the kit used.

Mineral analyses were performed by mineralization of 350 mg of each sample in closed vessels with nitric acid in a microwave digestion system (Ethos 1600 Milestone S.r.l., Sorisole, Bergamo, Italy). After dilution in ultrapure water to obtain a volume of 25 mL, the concentration of minerals was quantified by inductively coupled plasma optical emission spectrometry Ciros Vision EOP (Spectro Analytical Instruments GmbH, Kleve, Germany); Ca was determined at 317.933 nm, while the other minerals were determined as follows: P at 178.287 nm, Mg at 285.213 nm, Na at 589.592 nm, K at 766.941 nm, S at 182.034 nm, Al at 167.078 nm, B at 249.677 nm, Ba at 455.404 nm, Cr at 267.716 nm, Cu at 324.754 nm, Fe at 259.941 nm, Li at 670.780 nm, Mn at 257.611 nm, Mo at 202.095 nm, Ni at 231.604 nm, Sr at 407.771 nm, V at 292.464 nm, and Zn at 213.856 nm [24]. All the minerals were above the limit of detection of the instrument (0.01 ppm), with the exception of 13 samples for B and 11 samples for V. The fiber fractions were analyzed according to official methods 2002.04 for NDF, and 973.18 for ADF and ADL [25].

Total starch was quantified by an internal method of the laboratory following the directions of the Association of Official Analytical Chemists (AOAC) [25]. In brief, after weighing 500 mg of sample in a 100-mL PYREX glass tube (SciLabware, Stoke on Trent, United Kingdom), 50 mL of KOH (Carlo Erba, Milano, Italy) 0.5 M were added, and the mixture was stirred in a vortex mixer, heated in an oscillating water bath at 60 °C for 60 min, and cooled at room temperature. Subsequently, to reach a pH between 4.6 and 4.8, glacial acetic acid (Carlo Erba, Milano, Italy) was added, by pipetting, to the solution. Amyloglucosidase solution was prepared by pouring in a 50-mL PYREX glass flask (Schott Duran; Wertheim, Germany) 22.5 mg of amyloglucosidase standard from *Aspergillus niger* (10115, Sigma-Aldrich, Steinheim, Gemany) and making up to volume with deionized water (Millipore Corporation, Burlington, MA, USA). Thereafter, the sample solution with KOH and glacial acetic acid was added with 2 mL of amyloglucosidase solution and incubated overnight at 40 °C. After cooling at room temperature, the solution was diluted with deionized water to 100 mL; then, an aliquot of 10 mL was transferred to a 10-mL glass tube, centrifuged at 2700× *g* for 10 min, and the supernatant filtered through a 0.45 µm filter. For the quantification of glucose, 10 µL of the filtered supernatant were injected in a high-performance liquid chromatography spectra system equipped with Aminex HPX 87H column (Bio-Rad, Hercules, CA, USA) using an aqueous solution of sulfuric acid 0.0025 N as mobile phase and working conditions of a 0.6 mL/min flow rate and 38 °C internal temperature; a glucose standard solution as well as a calibration line were prepared. For the glucose standard solution, 200 mg of D-(+)-glucose anhydrous (G-7528, Sigma-Aldrich, Steinheim, Germany) were weighed in a 50-mL glass flask and made up to volume with deionized water. Subsequently, a known aliquot of the glucose standard solution for each calibration level was transferred to a 25-mL flask and made up to volume with sulfuric acid 0.1 N. Starch quantification was performed in duplicate.

Gelatinized starch was quantified as described by İnal et al. [26], using a Starch damage assay kit (Megazyme Intl. Ireland Ltd., Co. Wicklow, Ireland) that follows the method 76-31.01 of the American Association of Cereal Chemists. Briefly, the method consists in the hydration and hydrolyzation of damaged starch granules to maltosaccharides and dextrins through a controlled treatment with purified fungal α-amylase; afterward, a treatment with purified amyloglucosidase degrades starch-derived dextrins to glucose, which is measured with a high-purity glucose oxidase/peroxidase reagent mixture.

### 2.3. Near-Infrared Spectroscopy Analysis

Spectra were recorded using SCiO™ (Consumer Physics Inc., Tel Aviv, Israel), the handheld web-based wireless instrument that operates in reflectance mode scanning the NIR region, in the wavenumber range between 740 and 1070 nm (13,514 and 9346 cm ^−1^), at intervals of 1 nm (13 cm ^−1^). To reduce the incidence of anomalous reflectance values due to the heterogeneity of the surface caused by the casual arrangement of the product, spectra, which contained 331 data points, were determined in each sample 10 (intact sample) or 5 times (ground samples). Recordings were performed on 50 g of kibble placed in a plastic bag by applying the scanning head at 1 cm over the surface at different points. Then, spectra were collected through Mosaic software (FOSS, Hillerød, Denmark), converted to absorbance as log(1/reflectance), and each sample replicate was averaged for the subsequent development of NIRS prediction models.

### 2.4. Chemometric Analysis

The chemometric analysis was performed using WinISI 4 software (Infrasoft International, Port Matilda, PA, USA). Prediction models for total and gelatinized starch, and for the fibrous fractions were developed through modified partial least squares regression analysis performing both a leave-one-out cross-validation and an external validation, whereas prediction models for macrominerals and trace minerals were developed only performing cross-validation due to the limited number of samples. The leave-one-out cross-validation uses the entire dataset and excludes a single sample in each iteration; the external validation used a subset of 75% of samples of the entire dataset as a calibration set to produce a prediction equation to be tested on the remaining 25% of samples. To create the two subsets, the full dataset was randomly divided, ensuring a similar mean and standard deviation for each trait.

In order to increase the accuracy of the calibration, three passes of outliers’ elimination were done setting the critical *T*-statistic value for *T* outliers detection to 2.5 standard error. Thus, samples with a predicted value larger than 2.5 standard error of cross-validation from the respective reference value were removed. The data underwent some combinations of scattering corrections (NONE, no correction; D, detrending; SNV, standard normal variate; SNV+D, standard normal variate and detrending; MSC, multiplicative scatter correction) and several derivative mathematical treatments (0,0,1,1; 1,4,4,1; 1,8,8,1; 2,5,5,1; 2,10,10,1; where the first digit is the number of the derivative, the second is the gap over which the derivative is calculated, the third is the number of data points in the first smoothing, and the fourth is the number of data points in the second smoothing [27]). The determination of the best models was based on the following: the number of latent factors (LF); the standard error of calibration (SE_C_), cross-validation (SE_CrV_), and external validation (SE_P_); the coefficient of determination of calibration (R^2^_C_), cross-validation (R^2^_CrV_), and external validation (R^2^_ExV_); and the residual predictive deviation of cross-validation (RPD) and external validation (RPD_ExV_). The RPD interpretation was based on Williams [28], who indicated the possible application of calibration models according to the RPD value obtained. Briefly, the application of the model is not recommended if the RPD is below 1.9; the prediction model can be used for screening if RPD ranges from 2.0 to 2.9, whereas it is considered good for quality control with RPD between 3.0 and 4.0, and excellent for any application if it is greater than 4.0. Residuals of prediction equations were normally distributed, and bias did not differ significantly from zero.

## 3. Results

### 3.1. Chemical Composition

Total starch content averaged 32.26 ± 7.15% (as fed), while the mean gelatinized starch content was 21.69 ± 5.86%, leading to a ratio between gelatinized and total starch of 0.67 ± 0.11 (Table 2). The insoluble fibrous fractions content was 16.01 ± 6.07%, 4.27 ± 2.36%, and 1.66 ± 0.76% for NDF, ADF, and ADL, respectively; cellulose content, which was calculated as the difference between ADF and ADL, averaged 2.61 ± 1.79%, and hemicellulose content, which was calculated as the difference between NDF and ADF, was on average 11.74 ± 4.80%. The most abundant macromineral was Ca (1.37 ± 0.47%) followed by P (0.99 ± 0.29%), whereas the most abundant trace mineral was Fe (344.73 ± 94.09 mg/kg). All components had a coefficient of variation greater than 15%, and the greatest values were observed for minerals, except for Mg, and fibrous fractions.

### 3.2. Handheld Near-Infrared Prediction Models

**Figure 1:**
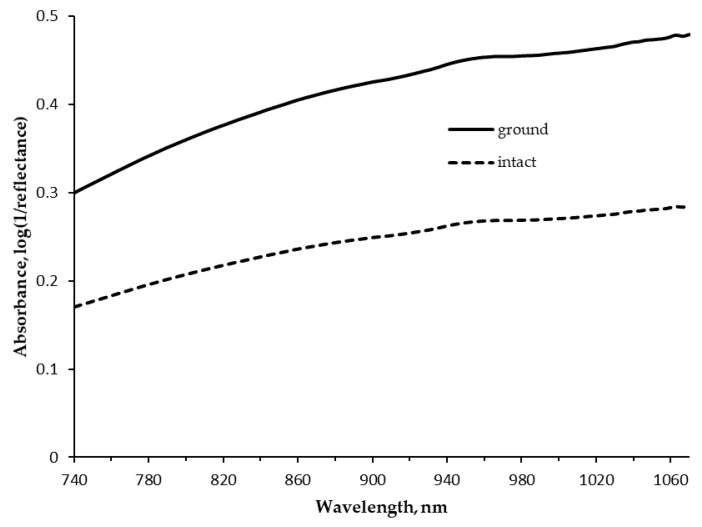
Average raw spectra of ground and intact dry dog food from a SCiO™ spectrometer.

The average raw absorbance spectra (log(1/reflectance)) of ground and intact samples are depicted in Figure 1. Absorbance values followed a similar increasing pattern for both sample treatments moving from 740 to 1070 nm but were greater for ground than intact samples. No clear peaks were detected across wavelengths, except for a wide and slight peak at 952 nm and a small peak at 1058 nm.

The performance of NIRS prediction models with R^2^_CrV_ ≥ 0.40 developed using leave-one-out cross-validation for starch, fibrous fractions, and minerals of ground and intact samples are reported in Table 3. Outliers detected in ground samples were ≤8%, except for NDF (12%) and Ni (10%), and LF ranged from 4 (Ni) to 10 (S). Regarding the intact samples, outliers were ≤11% and LF ranged from 3 (Li) to 8 (NDF). In ground samples, the best prediction models were obtained for gelatinized starch (RPD = 2.54; R^2^_CrV_ = 0.84) and total starch (RPD = 2.33; R^2^_CrV_ = 0.81), whereas insoluble fibrous fractions and minerals exhibited lower accuracies (RPD < 1.70; R^2^_CrV_ < 0.65), with the only exception of S (RPD = 1.92; R^2^_CrV_ = 0.72). In intact samples, the best calibration models were generated for the same traits (i.e., total and gelatinized starch) but with slightly lower accuracies for gelatinized starch (RPD = 2.45; R^2^_CrV_ = 0.83) and total starch (RPD = 2.08; R^2^_CrV_ = 0.77), and rather higher accuracies for insoluble fibrous fractions (RPD < 1.90; R^2^_CrV_ ≤ 0.71). The mineral with the most accurate prediction model in intact samples was K (RPD = 1.98; R^2^_CrV_ = 0.74). The results of external validation (Table 4) confirmed those obtained in cross-validation, with the highest accuracy for gelatinized starch in both ground (RPD = 2.55; R^2^_ExV_ = 0.89) and intact samples (RPD = 2.03; R^2^_ExV_ = 0.78). In ground kibbles, the lowest prediction accuracy was obtained for NDF, followed by ADL and ADF, whereas in intact kibbles, the lowest prediction accuracy was observed for ADF, followed by NDF and ADL. Overall, the best results were achieved with no scatter correction (NONE) or D, and first or second derivative as mathematical pre-treatment. Due to the unsatisfactory results, statistics of external validation for minerals were not reported.

## 4. Discussion

### 4.1. Chemical Composition Stated in the Label

Dog foods are produced mainly by international companies whose formulations are based on worldwide recognized guidelines (e.g., National Research Council (NRC) [8]), so their nutritional characteristics are expected to be similar all over the world. The gross composition reported by the manufacturer, as shown in Table 1, was confirmed by the data obtained with the owned FOSS prediction models (FOSS, Hillerød, Denmark) for dry pet food installed in NIRS DS2500 desktop instrument [15], which were on average 94.17 ± 1.42%, 31.54 ± 4.76%, 13.99 ± 2.78%, 3.59 ± 1.72%, and 7.06 ± 1.62% for dry matter (DM), and on a DM basis for crude protein (CP), ether extract (EE), crude fiber (CF), and ash, respectively. The DM and EE reported on the food labels and checked by desktop NIRS DS2500 were consistent with the values reported in the NRC [8]. The maximum values of CF and CP reported on food labels exceeded the range reported in the cited guidelines (2.5–10 and 18–32% DM for CF and CP, respectively). However, the composition results determined with the desktop NIRS DS2500 were more similar to the range reported in the regulation. No indications were reported for crude ash in the guidelines.

### 4.2. Chemical Composition and Handheld NIRS Evaluation

Several studies on commercial dry dog foods reported similar values for starch content [1,29]. The ratio of gelatinized to total starch was slightly lower than the ratio reported by Tran et al. [30] for dry extruded canine diets, which was probably because of different processing conditions or the larger amount of fat in the samples used in the present study before the thermal process, which decreased the degree of starch gelatinization and digestibility [31]. Given the RPD of prediction models developed for total and gelatinized starch, the handheld NIRS device can be successfully employed for rough screening purposes. Particularly, the prediction performed on ground samples seemed to be more accurate compared to that on intact kibbles.

The overall amount of fibrous fractions was greater than results from other studies. In particular, the average NDF was greater than the value reported by De-Oliveira et al. [10] for six dry dog foods; this is probably related to the wider variety of food included in the present study. It is worth highlighting that the knowledge of NDF is important because it is composed by insoluble and non-viscous components of plant fiber, which can affect the transit time and fecal consistency. The average NDF was in the range reported also by Sallander et al. [32], who obtained NDF between 6.7% and 19.4% DM. The ADF content was in agreement with the results of Tran et al. [30] and De-Oliveira et al. [10], and the average ADL was greater than the ADL observed by Opitz et al. [33]. Moreover, a greater variability was quantified in the present study compared to Opitz et al. [33], whose results ranged from 1.2 to 15.5 g/kg on DM. The cellulose content was in the range reported by Weber et al. [1]. In the latter study, large dog breeds had a positive reaction (i.e., higher fecal score) to the inclusion of higher level of cellulose in the diets, while small breeds can tolerate lower levels of cellulose. Indeed, diets with cellulose concentration >1.5% can result in constipation and a decrease of the stool quality [1]. The type of fiber fed to the dog affects the moisture content of the feces. In fact, feces of dogs fed with pectin or guar gum, which are viscous plant fiber, are softer than those from dogs fed a diet containing cellulose, which has lower hydration capacity. Furthermore, a linear decrease in mean retention time of digesta was associated to the increased beet pulp concentration [7]. Beet pulp and wheat bran are considered moderately fermentable fiber sources due to their high content of NDF (particularly hemicellulose), which affects transit time, stool weight, moisture, and composition [8]. Despite the positive effects of these two foods, there is a paucity of information in the literature regarding the effects of the hemicellulose per se. Moreover, it has been demonstrated that crude fiber can play a role in the management of paraphysiological or pathological conditions, such as obesity and diabetes that are nowadays emerging diseases related to both inaccurate diets formulation and aging [34]. The type of fiber fed to dogs can have different energy content, and the unfermentable and insoluble fiber can reduce the energy density of the diet [8].

The macro and trace mineral content chemically analyzed corresponded or slightly exceed (without overcame the safe upper limit) the recommended doses reported by the NRC [8]. An extreme value was found for Ca, which exceeded the recommended amount for healthy animals. It should be noted that the excess of Ca in animals under physiological conditions lowers the parathyroid gland activity, inducing bone lesions [14]. The content of Fe, Zn, Mn, Cu, and Cr is similar to those recommended [8] and reported in the literature [13,14,35,36]. Despite the paucity of information to indicate the safe upper limit of many macro- and trace minerals, their over supplementation can cause several problems as urolithiasis, mesenteric venoconstriction (Na), hypertension (salt excess), vomiting (Ni), diarrhea, gastrointestinal bleeding, and also toxicity (Fe, Sr), chronic hepatitis and cirrhosis (Cu). Additionally, Mg over supplementation needs to be avoided in dogs with renal failure, while K overfeeding is not indicated for dogs affected by cardiac diseases or chronic kidney disease. Moreover, deficiency in some minerals and vitamins increases the susceptibility to abnormalities in hair growth, reduces serum levels of thyroid hormones (Se), causes skin diseases, and increases the prevalence of infections [8,14]. The low RDP values obtained for the macro- and microminerals content analyzed by the handheld NIRS indicated the models’ poor accuracy of prediction. Two exceptions were found for the S content evaluated on the ground kibbles and K content evaluated on the intact kibbles, but still, based on the RPD, the predictive ability of NIRS also for these minerals can be considered insufficient [28].

### 4.3. Near Infrared Spectrum

The average raw spectrum (Figure 1) is consistent with the meat spectra reported by Kademi et al. [18] and Cozzolino and Murray [37], who reported the same trend without noticeable peaks in the same range of wavelengths, which was probably due to the weakness of all the bands that appeared in the region between 800 and 1200 nm [38]. The flat peak observed around 952 nm can probably arise from the second overtone of O–H stretching of water interacting with protein, even if the region of 950 to 960 nm could also have some relationship to fat–water interaction as reported in NIR studies on milk and salmon [39]. Furthermore, in agreement with Williams and Norris [40], NIR absorbance bands for cellulose can be identified at 1058 nm. The difference observed in the absorbance values of ground and intact kibbles, also reported by Cozzolino et al. [41] in pork muscles, might be due to the variation of particles aggregation and to the light scattering from the sample, but it manifested itself only with a change in the height of the average spectral profile but not in the shape. Despite the positive aspects of the handheld SCiO™ instrument, which are the basis for companies’ decision to adopt this new tool, it is necessary to highlight that the spectral range in which it operates is limited. This can ensure the cost competitiveness but lead to a lack of absorption signals related to the presence of carbohydrates, starch, or other possible components that could be detected by desktop NIRS instruments [42], and it probably limits the predictive capacity for complex nutritional parameters [43].

### 4.4. Prediction Models

In general, the quantitative prediction of total and gelatinized starch obtained by cross-validation can be considered good for a rough screening ([28]; RPD_CrV_ > 2.0), and the sample preparation (i.e., intact or ground) provided similar calibration statistics for those compounds (Table 3). When the accuracy of prediction models was tested in an independent dataset through the external validation, the prediction of total starch was considered not satisfactory, with RPD_ExV_ of 1.75 and 1.89 for ground and intact samples, respectively; the prediction of gelatinized starch was fairly good for screening in ground samples (RPD_ExV_ = 2.55) and good for an approximate screening in intact samples (RPD_ExV_ = 2.03; Table 4). The literature on the prediction of total and gelatinized starch in pet food is very scarce; nevertheless, the pet food industry considers these parameters very informative to monitor the cooking and production processes. Prediction models for fibrous fractions did not reach a satisfactory accuracy (RPD_CrV_ < 1.9), which was most likely because of the small quantities of these components and the narrow range of wavelengths in which the instrument works. However, studies performed using laboratory instruments with a greater light source and operating in a wider range of wavelengths revealed the good prediction ability of NIRS for fibrous fractions [44,45,46]. Similarly, poor results were obtained for minerals. According to Karoui et al. [47], prediction models developed with cross-validation could be adequate for approximate quantitative prediction (R^2^_CrV_ > 0.66) for S in ground samples and for K in intact samples. No satisfactory models were obtained for any other mineral tested (Ca, P, Mg, Na, Al, B, Ba, Cr, Cu, Fe, Li, Mn, Mo, Ni, Sr, V, and Zn) using both cross- and external validation. This can be explained because, unlike organic molecules, minerals can be detected if chelated in organic complexes or indirectly if they have an effect on hydrogen bonds [48] and often, even if minerals are added to pet food in order to meet animals’ requirements, their form is inorganic, and therefore, they are less likely to be identified and well predicted. However, NIRS technology has been used in several studies to evaluate the mineral content in food matrices [49], and to the best of our knowledge, only Alomar et al. [36] and Goi et al. [40] have assessed the prediction ability in dry pet food using NIRS laboratory instruments, but no studies on dry dog food have been conducted considering the narrow range of wavebands of the present research.

## 5. Conclusions

The present study suggests that total and gelatinized starch can be successfully predicted using the handheld SCiO™ instrument; this opens the opportunity to monitor their content and ratio in commercial dog food on a large-scale. However, the instrument is not adequate to predict insoluble fibrous fractions as well as mineral content, except for S and K, and in general to perform quality and process controls. The miniaturization of the NIR instruments is an advantage for the pet food industry to perform fast inspections along the production chain, but to date, the performance allows only a rough evaluation of some components; improvement, such as widening the wavelength range would likely increase its accuracy. Unlike other tools, the handheld miniaturized infrared spectrometer could be used in those factories where the economic investment required to install NIR instruments for at-line predictions is not affordable.

## Figures and Tables

**Table 1:** Chemical composition of the commercial dry dog food samples (*n* = 99) included in the study reported by the manufacturer and expressed on a dry matter basis (% dry matter, DM).

Item	Mean	SD	Minimum	Maximum	CV
DM	92.00	0.00	92.00	92.00	0.0
Crude protein	30.27	4.95	23.91	41.30	16.4
Ether extract	16.87	3.60	10.33	21.74	21.3
Crude ash	7.24	1.26	2.50	9.78	17.5
Crude fibers	3.58	2.29	2.28	15.22	63.8
Nitrogen-free extract	41.75	8.06	25.54	54.89	19.3

CV = coefficient of variation; %; Nitrogen-free extract: 100 − (crude protein + ether extract + ash + crude fiber); SD = standard deviation.

**Table 2:** Chemical composition (as-fed) from lab chemical reference analyses of dry dog food samples.

Item	*n*	Mean	SD	Minimum	Maximum	CV
Starch, %						
Total	81	32.26	7.15	11.50	43.23	22.2
Gelatinized	81	21.69	5.86	9.15	35.00	27.0
Insoluble fiber, %						
NDF	99	16.01	6.07	7.74	36.55	37.9
ADF	99	4.27	2.36	1.98	16.84	55.4
ADL	99	1.66	0.76	0.41	4.34	45.5
Cellulose	99	2.61	1.79	1.06	12.54	68.6
Hemicellulose	99	11.74	4.80	5.19	28.19	40.9
Macrominerals, %						
Ca	99	1.37	0.47	0.44	3.58	34.4
P	99	0.99	0.29	0.35	1.84	29.5
K	99	0.70	0.31	0.28	1.52	43.9
Na	99	0.51	0.17	0.12	0.91	33.4
S	99	0.38	0.13	0.20	0.79	33.6
Mg	99	0.11	0.02	0.08	1.50	15.3
Trace minerals, mg/kg						
Fe	99	344.31	86.94	121.68	658.52	25.2
Zn	99	179.44	53.14	35.11	307.39	29.6
Al	99	152.88	51.38	51.73	288.23	33.6
Mn	99	66.66	23.00	14.15	230.75	34.5
Cu	99	22.67	5.47	9.34	43.80	24.1
Sr	99	17.83	9.62	5.68	64.23	53.9
Ba	99	5.41	2.17	1.39	13.64	40.1
B	86	4.52	1.95	0.99	9.77	43.2
Cr	99	1.99	1.06	0.59	8.38	53.2
Ni	99	1.21	0.34	0.52	2.45	27.8
Mo	99	0.67	0.20	0.23	1.29	29.8
V	88	0.38	0.24	0.13	1.43	64.4
Li	99	0.19	0.09	0.08	0.59	46.7

ADF = acid detergent fiber; ADL = acid detergent lignin; NDF = neutral detergent fiber; Cellulose = ADF − ADL; Hemicellulose = NDF − ADF.

**Table 3:** Fitting statistics of modified partial least squares regression models in leave-one-out cross-validation for total and gelatinized starch, NDF, ADF, ADL (% as-fed), and minerals content^1^ in ground and intact dry dog food.

Item	*n*	LF	Mean	SD	R^2^_C_	SE_C_	R^2^_CrV_	SE_CrV_	RPD
Ground kibbles									
Total starch	75	6	32.89	6.54	0.91	1.91	0.81	2.80	2.33
Gelatinized starch	76	7	22.00	5.78	0.87	2.08	0.84	2.27	2.54
NDF	87	6	15.60	5.15	0.71	2.78	0.56	3.39	1.52
ADF	93	6	3.84	1.23	0.57	0.80	0.45	0.91	1.35
ADL	93	5	1.57	0.62	0.76	0.30	0.64	0.37	1.68
Cellulose	86	7	2.23	0.78	0.56	0.52	0.39	0.61	1.28
Hemicellulose	94	9	11.72	4.67	0.73	2.41	0.58	3.00	1.56
K	94	7	0.67	0.29	0.60	0.18	0.56	0.19	1.51
Na	93	8	0.51	0.16	0.67	0.09	0.56	0.11	1.53
S	92	10	0.37	0.12	0.85	0.04	0.72	0.06	1.92
Mg	94	6	0.11	0.02	0.68	0.01	0.55	0.01	1.45
Ni, mg/kg	89	4	1.16	0.29	0.60	0.18	0.41	0.22	1.32
V, mg/kg	83	5	0.35	0.20	0.69	0.11	0.44	0.15	1.33
Li, mg/kg	91	5	0.18	0.06	0.60	0.04	0.51	0.04	1.50
Intact kibbles									
Total starch	72	7	32.27	6.43	0.89	2.13	0.77	3.09	2.08
Gelatinized starch	74	5	21.55	5.69	0.89	1.89	0.83	2.32	2.45
NDF	94	8	15.42	5.26	0.78	2.49	0.61	3.27	1.61
ADF	91	7	3.77	1.15	0.69	0.64	0.56	0.76	1.51
ADL	91	6	1.56	0.61	0.78	0.29	0.71	0.33	1.86
Cellulose	90	8	2.22	0.74	0.59	0.47	0.44	0.54	1.37
Hemicellulose	95	8	11.64	4.73	0.83	1.98	0.63	2.86	1.65
K	92	5	0.67	0.29	0.80	0.13	0.74	0.15	1.98
Na	95	4	0.52	0.17	0.65	0.10	0.47	0.12	1.38
S	92	5	0.37	0.12	0.69	0.07	0.62	0.07	1.61
Mg	95	6	0.11	0.02	0.64	0.01	0.55	0.01	1.55
Sr, mg/kg	95	5	16.52	6.99	0.53	4.81	0.40	5.40	1.29
Cr, mg/kg	93	5	1.85	0.70	0.61	0.44	0.48	0.51	1.37
Ni, mg/kg	94	7	1.17	0.29	0.65	0.17	0.43	0.22	1.32
Li, mg/kg	95	3	0.18	0.06	0.56	0.04	0.47	0.05	1.20

^1^ Minerals’ predictions with R^2^_CrV_ < 0.40 have not been reported. LF = optimal number of latent factors, *n* = number of samples; R^2^_C_ = coefficient of determination of calibration, R^2^_CrV_ = coefficient of determination of cross-validation, RPD_CrV_ = residual predictive deviation of cross-validation, SD = standard deviation, SE_C_ = standard error of calibration, and SE_CrV_ = standard error of cross-validation.

**Table 4:** Fitting statistics of modified partial least squares regression models in external validation for total and gelatinized starch, and NDF, ADF, and ADL (% as fed) in ground and intact dry dog food.

Item	Calibration set ^1^			Validation set ^2^				
	*n*	SE_CrV_	R^2^_CrV_	Bias	Slope	SE_P_	R^2^_ExV_	RPD_ExV_
Ground kibbles								
Total starch	55	3.05	0.76	0.07	0.87	3.17	0.69	1.75
Gelatinized starch	54	2.33	0.81	−0.59	0.82	2.49	0.89	2.55
NDF	67	3.20	0.53	0.90	0.79	3.13	0.56	1.45
ADF	67	0.87	0.43	0.03	1.27	0.74	0.61	1.55
ADL	61	0.37	0.65	−0.01	0.74	0.43	0.62	1.48
Intact kibbles								
Total starch	56	3.19	0.77	−0.31	0.95	3.19	0.72	1.89
Gelatinized starch	54	2.28	0.81	−0.14	0.84	3.14	0.78	2.03
NDF	69	3.49	0.49	0.30	1.00	3.58	0.62	1.61
ADF	68	0.78	0.58	0.02	0.96	0.86	0.52	1.44
ADL	69	0.36	0.66	−0.09	0.79	0.34	0.70	1.69

^1^ 75% of the entire dataset (*n* = 60 for total and gelatinized starch; *n* = 74 for fibrous fractions). ^2^ 25% of the entire dataset (*n* = 21 for total and gelatinized starch; *n* = 25 for fibrous fractions). *n* = number of samples of the calibration dataset, excluding the outliers, with which the calibration curve was developed.
